# Short communication: Dynamic chemical labelling allows the measurement of the paracetamol toxicity biomarker microRNA‐122 in hospital clinical laboratories

**DOI:** 10.1002/bcp.70364

**Published:** 2025-11-17

**Authors:** Samar Alzeer, Antonio Marin‐Romero, Bárbara López‐Longarela, Juan J. Guardia‐Monteagudo, F. Javier Lopez‐Delgado, Salvatore Pernagallo, Juan J. Diaz‐Mochon, Mavys Tabraue‐Chavez, Kathleen Scullion, James W. Dear

**Affiliations:** ^1^ Centre for Cardiovascular Science, Queen's Medical Research Institute University of Edinburgh Edinburgh UK; ^2^ DESTINA Genomica S.L. Parque Tecnológico Ciencias de la Salud (PTS) Granada Spain; ^3^ GENYO Centre for Genomics and Oncological Research, Pfizer/University of Granada/Andalusian Regional Government Granada Spain; ^4^ Department of Medicinal and Organic Chemistry, Faculty of Pharmacy University of Granada Granada Spain; ^5^ Unit of Excellence in Chemistry Applied to Biomedicine and the Environment of the University of Granada Granada Spain; ^6^ Instituto de Investigación Biosanitaria ibs. GRANADA Granada Spain; ^7^ Present address: Vircell S.L. Parque Tecnológico de la Salud Granada Spain

**Keywords:** dynamic chemical labelling, miR‐122, multiplex immunoassay, paracetamol toxicity

## Abstract

MicroRNA‐122 (miR‐122) is a sensitive biomarker for paracetamol‐induced liver injury. This study assessed the diagnostic performance of dynamic chemical labelling (DCL) combined with an immunoassay platform to quantify miR‐122 in human serum, as a proof‐of‐concept for hospital use. This study used serum samples from 19 healthy individuals and 19 patients with paracetamol drug‐induced liver injury (DILI) collected in previous studies. miR‐122 was labelled with a biotinylated SMART‐Base and quantified using a multiplex, bead‐based, fluorescence detection system. The median miR‐122 concentration was significantly elevated at 233.4 pg/mL (IQR: 74.5–363.4) compared to 15.2 pg/mL (IQR: 9.4–24) in the control group. The Mann–Whitney *U* test showed significant group differences (*p* < 0.0001). ROC analysis yielded an area under the curve (AUC) of 0.96, with a 29.7 pg/mL cut‐off providing 92.6% sensitivity (95% CI: 76.6%–98.7%) and 89.4% specificity (95% CI: 68.6%–98.1%). miR‐122 moderately correlated with ALT (*r* = 0.56, *p* = 0.03). DCL with fluorescence detection is a promising clinical tool for miR‐122 quantification.

**What is already known about this subject**

- Paracetamol overdose is a leading cause of acute liver failure.
- Liver function biomarkers such as ALT are commonly used to assess toxicity.
- miR‐122 is a promising sensitive and specific biomarker for liver injury.

**What the study adds**

- Dynamic chemical labelling enables the direct measurement of miR‐122 in serum without using quantitative PCR.
- The multiplex bead‐based system used for detection is available in hospital laboratories, making the assay accessible for clinical use.
- This study demonstrates the feasibility of miR‐122 quantification in paracetamol overdose, supporting future research into its clinical utility.

## 1 INTRODUCTION

Paracetamol overdose is the leading cause of acute liver failure in Western countries.^1^, ^2^ The prompt administration of the antidote N acetylcysteine (NAC) to patients at risk of liver injury is crucial to prevent potentially life‐threatening liver failure. Treatment efficacy decreases substantially as the delay between overdose and starting NAC increases. NAC is almost 100% effective if started within 8 h of overdose, but efficacy decreases when treatment is delayed beyond this time window.^3^

There are no specific symptoms or clinical signs of early liver damage. Therefore, healthcare workers rely on blood tests to detect liver injury after overdose. However, the standard serum biomarker for liver injury diagnosis, alanine aminotransferase (ALT), increases too slowly post‐overdose to accurately diagnose early liver injury within the NAC optimal therapeutic window. Therefore, the current method for early risk stratification after paracetamol overdose is largely dependent on the patient‐reported time of overdose to estimate the delay between overdose and NAC. This approach is inherently subjective, cannot be used in staggered overdoses and accidental therapeutic excess ingestions (together accounting for around one third of cases in UK) and has suboptimal accuracy even when applied correctly. There is a pressing need for a rapid, cost‐effective, point‐of‐care assay to identify high‐risk patients at hospital presentation (or even in the ambulance pre‐hospital) with sufficient sensitivity and specificity for targeted early treatment.

Circulating microRNA‐122 (miR‐122), a highly liver‐specific microRNA, has emerged as a biomarker for paracetamol‐induced liver injury, demonstrating rapid and significant increases in serum within hours of toxic paracetamol exposure; importantly preceding ALT elevation.^4^, ^5^, ^6^, ^7^, ^8^ While these, and other studies, have validated the sensitivity and specificity of miR‐122, their reliance on quantitative PCR (qPCR) methods has hindered clinical translation. Although highly sensitive, qPCR requires specialized equipment, trained personnel and relatively lengthy processing times (typically several hours), making it unsuitable for the time‐critical decision‐making required in acute paracetamol overdose management.^6^, ^9^ Previous studies have demonstrated the feasibility of using dynamic chemical labelling (DCL) technology for miR‐122 quantification.^10^, ^11^, ^12^, ^13^ However, these studies utilized a highly specialized, non–in‐vitro diagnostic (IVD)‐certified single‐molecule array (Simoa) platform,^14^ which is not readily available in clinical laboratories. Therefore, a rapid, clinically accessible and IVD‐compatible assay for miR‐122 to aid in the management of acute paracetamol overdose remains an unmet need.

To address this need, we have developed a novel miR‐122 immunoassay‐like assay combining DCL with the Luminex xMAP platform. The Luminex xMAP technology is a bead‐based multiplex immunoassay platform that is already widely used in clinical laboratories worldwide for a variety of diagnostic applications, including infectious disease testing and HLA typing.^15^ There are reportedly more than 18 000 installed Luminex xMAP platforms, of which an estimated 25%–30% are in clinical laboratories. Therefore, this ‘Luminex‐DCL’ miR‐122 assay leverages existing hospital infrastructure, eliminating the need for specialized equipment or extensive training, and offers a significantly reduced turnaround time compared to qPCR, potentially enabling results within approximately 1 h. The objective of this study was to explore the diagnostic performance of the Luminex‐DCL miR‐122 assay for detection of paracetamol‐induced liver injury in patients. We hypothesized that the serum miR‐122 concentration, measured by the Luminex‐DCL assay (index test), would be significantly elevated in patients with paracetamol‐induced liver injury (defined by the reference standard test—ALT), compared to healthy controls.

## 2 METHODS

### 2.1 Samples

A total of 38 serum samples were analysed to compare miR‐122 concentration in patients diagnosed with paracetamol DILI (*N* = 19) and healthy subjects (*N* = 19). Paracetamol DILI was defined as a serum ALT activity greater than 5× the upper limit of normal (ULN) after a patient reported paracetamol overdose which was confirmed by an elevated blood paracetamol concentration (ULN = 50 U/L). DILI samples were randomly selected from The Markers and Paracetamol Poisoning Study 2 (MAPP2, ClinicalTrials.gov identifier: NCT03497104). Ethical approval for this study was provided by London‐South East Research Ethics Committee (18/LO/0894), and all patients provided written informed consent. The study established a biobank of human serum samples from patients with paracetamol overdose. MAPP2 was a prospective, observational cohort study of participants presenting to the Emergency Department at the Royal Infirmary of Edinburgh, UK. Inclusion criteria were that participants must be age 16 years and over, attending hospital with a paracetamol overdose alone or as part of a mixed overdose and able to give informed consent. Healthy human serum was collected from healthy individuals on the Centre for Inflammation Research Blood Donor Register, University of Edinburgh (Ethics Reference Number: 21‐EMREC‐04). All healthy controls from the Blood Donor Registry provided informed consent for the use of their specimens in research, in accordance with ethical approval. In line with ethical requirements, only the participants' gender and age range were reported.

All samples were stored at −70°C until analysis. Before assay preparation, samples were thawed completely, vortex‐mixed thoroughly and centrifuged to ensure homogeneity and remove any debris. The processed samples were then used for downstream analysis following the assay protocol.

### 2.2 The DCL technology

**FIGURE 1:**
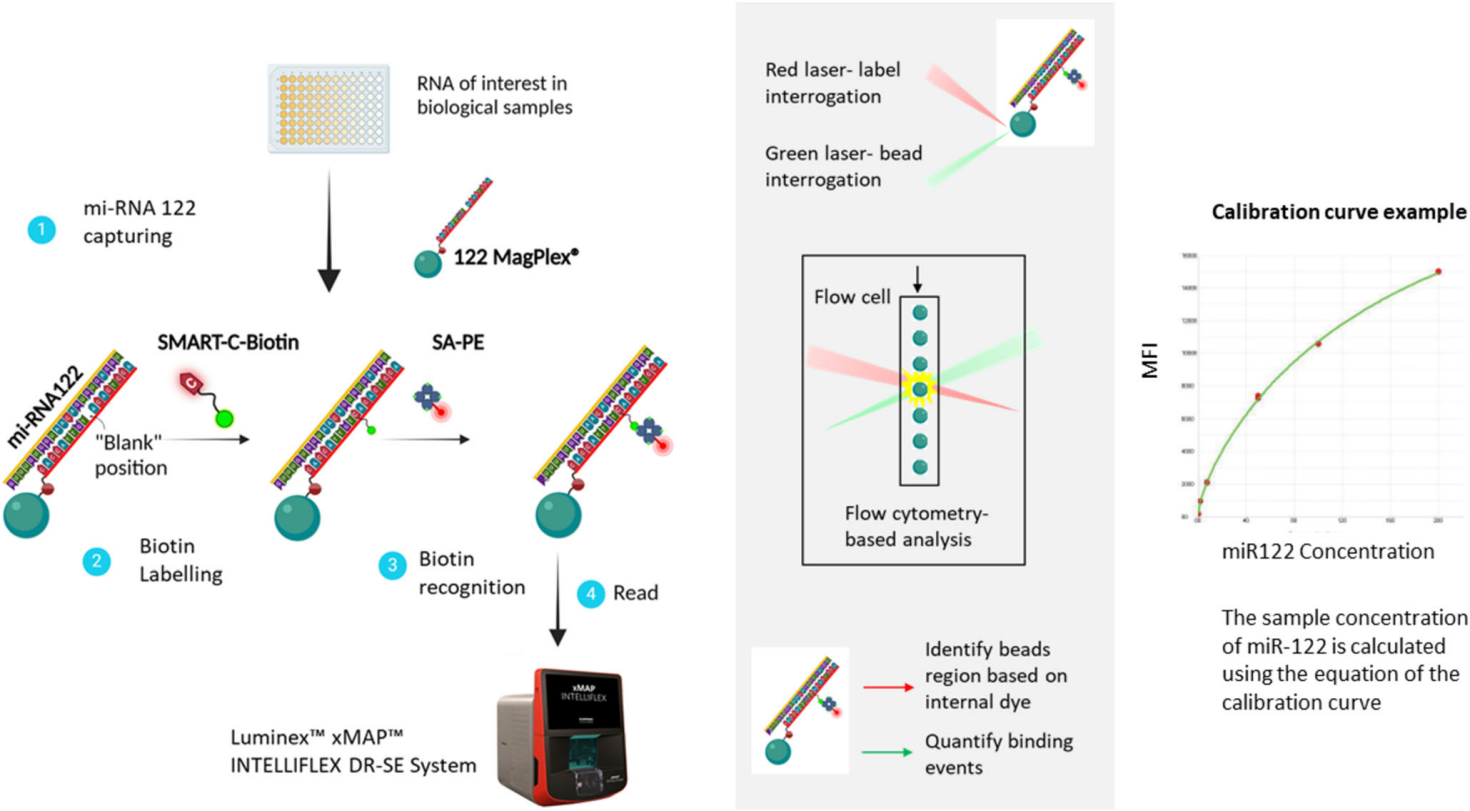
Schematic representation of a miRNA detection assay using dynamic chemistry labelling (DCL). 1. Samples are mixed with the beads and miR‐122 is captured by miR‐122 microsphere beads. 2. Biotin labelling is allowed because of dynamic chemistry labelling using a biotinylated SMART base. Only the beads with the captured miR‐122 will be labelled. 3. Biotin recognition by streptavidin‐Phycoerythrin (SA‐PE) enables fluorescent detection 4. Reading in xMAP™ INTELLIFLEX™ Luminex instrument where one laser classifies the bead type to determine the analyte that is being detected (used essentially to allow the multiplexing), while a second laser determines the magnitude of the bound analyte (PE‐derived signal). Calibration curve using miR‐122 standards allows miR‐122 quantification.

The Liver Injury miR‐122 Test (LiverACE™ Kit, LAK‐96‐2024) was provided by Destina Genomica SL (Granada, Spain). This assay is based on DCL Technology which combines miRNAs capture probes linked to MagPlex® Microspheres and a biotinylated SMART‐Base to selectively label duplex formation with the target miRNA sequence. Upon hybridization, the SMART‐Base incorporates into the miRNA‐probe duplex, allowing for specific chemical labelling. Detection was performed using a reporter molecule that binds to the biotin tag, generating a signal proportional to the miRNA concentration. The fluorescence signal was then analysed using the Luminex xMAP INTELLIFLEX DRSE System (Figure 1).

### 2.3 Microsphere‐based multiplexing technology

The Luminex xMAP INTELLIFLEX DRSE from ThermoFisher Scientific was used in this study. This technology combines flow cytometry with microsphere‐based assays, allowing for enhanced sensitivity, specificity and data precision. Data processing was refined using the Luminex xMAP INTELLIFLEX DRSE software, allowing for comprehensive signal integration and normalization. The Luminex System was configured according to the assay requirements. The probe height was adjusted based on the plate used to ensure optimal sample acquisition. In the acquisition Settings, the bead type was set to MagPlex®, with a minimum measurement of 50 events per bead. Each sample was analysed with a sample size of 100 μL, and the gate setting was adjusted between 8000 and 17 000 to capture the appropriate bead population. The reporter gain was set to Low PMT, with a timeout of 60 s to ensure adequate bead detection. The assay utilized singleplex bead sets, allowing for precise and reproducible analyte quantification.

### 2.4 Assay reaction

The Bio‐Plex Handheld Magnetic Washer (Bio‐Rad, 171 020 100) was used during the washing steps of the assay. The procedure was conducted following the manufacturer's instructions. A calibration curve was performed using seven serial dilutions of synthetic miR‐122 oligonucleotide to spike in an assay matrix. Concentrations of 20 000, 5000, 1250, 313, 78, 20 and 5 pg/mL were used and assay matrix without spike‐in was used as the blank sample. Briefly, 25 μL of test solution (either serum sample or calibrator) was added to a flat‐bottom 96‐well plate containing 75 μL of miR‐122 microsphere beads in each well. The plate was incubated on a plate shaker at 30°C for 2 h to allow binding of the analytes to the beads. After binding, the beads were washed three times with wash buffer. Subsequently, 50 μL of LiverAce™ Mix, containing SMART‐C and a Reducing Agent in Assay Buffer, was added to each well. The plate was shaken for 1 h at 40°C, followed by three additional washing steps. Then, 50 μL of Streptavidin‐R‐Phycoerythrin (SA‐PE) was added, and the plate was further incubated for 30 min at 30°C on a plate shaker. After a final series of three washes, the beads were resuspended in 120 μL of wash buffer and analysed on the Luminex xMAP INTELLIFLEX DRSE system. All the calibrators and samples were analysed in triplicate. The concentration of miR‐122 in samples was obtained with a curve interpolation using the GraphPad Prism 10 software.

### 2.5 Clinical biomarkers

Blood tests were measured in the NHS Lothian Biochemistry Laboratory as described previously.^5^

### 2.6 Statistical analysis

Data were statistically analysed with GraphPad Prism 10 software (La Jolla, California). Interquartile ranges (IQR) are presented for median values. For each sample, the reported miR‐122 value corresponds to the average of three measurements performed on the sample. The Mann–Whitney *U* tests were used for non‐parametric comparisons between groups. Receiver operating characteristic (ROC) analysis was conducted to evaluate the diagnostic performance of miR‐122 as a biomarker for liver injury, and the area under the curve (AUC) was calculated to determine sensitivity and specificity. The threshold was selected directly from the ROC analysis by identifying the cut‐off that provided the highest combined sensitivity and specificity. Statistical significance was set at *p* < 0.05 for all tests. Spearman's correlation coefficient was used to assess the relationship between miR‐122 and ALT concentrations.

### 2.7 Nomenclature of targets and ligands

Key protein targets and ligands in this article are hyperlinked to corresponding entries in http://www.guidetopharmacology.org and are permanently archived in the Concise Guide to PHARMACOLOGY 2021/22.^16^

## 3 RESULTS

The study included a total of 38 participants, with 19 healthy controls and 19 patients with paracetamol‐induced liver injury (DILI). The majority of healthy controls were aged 21–30 years (13 out of 19), followed by 41–50 years (5/19) and 31–40 years (1/19), and 10/19 were males.

Table 1 provides a detailed overview of the demographic and clinical characteristics of the patient cohort. The median paracetamol ingestion was 26 g (IQR: 16–46 g), and 68.4% of patients had co‐ingested other substances, primarily ethanol or opioids. Biochemical tests were performed to assess liver function, revealing elevated ALT activity (median: 558 U/L, IQR: 306–1301) in patients with liver toxicity. Additional biochemical parameters, including alkaline phosphatase, sodium, potassium, creatinine, urea, WBC, haemoglobin, and INR, are presented in Table 1.

**TABLE 1:** Participant characteristics and laboratory results for 19 participants with paracetamol induced liver injury.

Age years: Median (IQR)	30 (21–51)
Gender: Male (%)	4 (21)
Age: Median (IQR)	30 (21–51)
Ethnicity (%)	
White Scottish	18 (94.7%)
White (other)	1 (5.2%)
Overdose type (%)	
Acute overdose <8 h	7 (36.8%)
Acute overdose >8 h	8 (42.1%)
Supra‐therapeutic overdose	2 (10.5%)
Staggered intentional overdose (ingestion over 2 h or more)	2 (10.5%)
Total paracetamol ingested in grammes: Median (IQR)	26 (16.0–46.2)
Ingestion of other drugs: Yes (%)	13 (68.4%)
Anti‐coagulants	0
Non‐opioid analgesics	2
NSAIDs	2
Cardiovascular drugs	0
Alcohol	7
Opioids	6
SSRIs	1
Tricyclic antidepressants	1
Benzodiazepines	0
Other	1
Time of collection (hours from hospital presentation): median (IQR)	**27.1 (18.2–48.8)**
Prothrombin (secs): Median (IQR)	14.2 (13.1–17)
Alk Phos (U/L): Median (IQR)	69 (57–131)
Creatinine (μmol/L): Median (IQR)	56 (51–76.2)
Urea (mmol/L): Median (IQR)	2.9 (2.3–4.1)
WBC (x10^9^/L): Median (IQR)	8.8 (5.8–9.5)
Potassium (mmol/L): Median (IQR)	3.6 (3.1–3.8)
INR: Median (IQR)	1.3 (1.2–1.5)
ALT (U/L): Median (IQR)	558 (306–1301)
Haemoglobin (g/L): Median (IQR)	126 (114–137.2)
Sodium (mmol/L): Median (IQR)	139 (137.7–141.2)
Bilirubin (μmol/L): Median (IQR)	16.5 (10–32.7)

**FIGURE 2:**
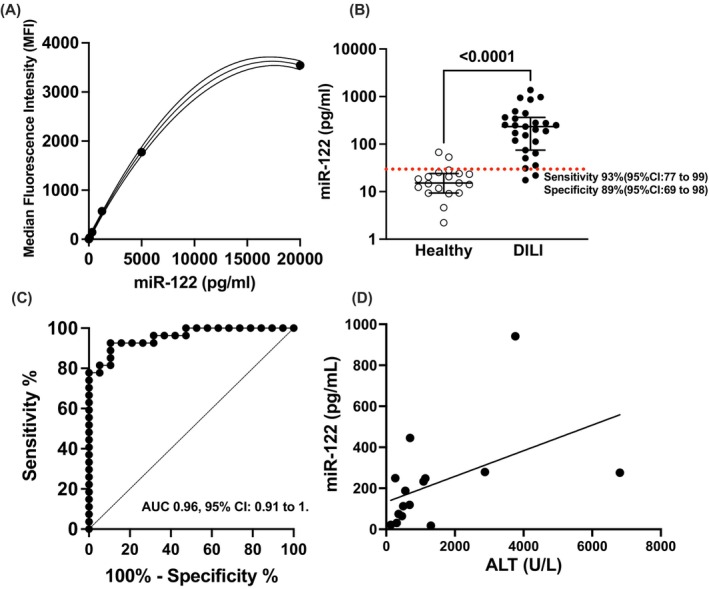
(A) Standard curve for miR‐122 quantification using microsphere‐based multiplex technology. The *x*‐axis represents miR‐122 concentration (pg/mL), while the y‐axis represents the median fluorescence intensity (MFI). (B) Serum miR‐122 concentration (pg/mL) in patients with paracetamol‐induced liver injury (DILI) and healthy controls. Values represent the mean of three independent measurements per sample. A significant difference in miR‐122 was observed between the two groups, as determined by the Mann–Whitney *p*‐value (*p* < 0.0001). The bars represent the median with error bars representing the IQR. Red dotted line represents sensitivity and specificity at a cut off value of 29.7 pg/mL. (C) Receiver operating characteristic (ROC) curve for miR‐122 in distinguishing paracetamol‐induced hepatotoxicity patients from healthy controls. The *X*‐axis represents 1—specificity (false positive rate, %), and the Y‐axis represents sensitivity (true positive rate). The area under the curve (AUC) is 0.96 (95% CI: 0.91 to 1). (D) Scatter plot depicting the correlation between circulating miR‐122 concentration (pg/mL) and alanine aminotransferase (ALT) activity (U/L) in patients with paracetamol‐induced liver injury. Each dot represents an individual sample. Spearman correlation analysis demonstrated a moderate positive correlation (*r* = 0.56, 95% CI: 0.056 to 0.84, *p* = 0.031). Black line represents the simple linear regression between miR‐122 and ALT.

Figure 2A shows a calibration curve of miR‐122 that was generated by plotting the fluorescence signal (MFI) against known miR‐122 concentrations (pg/mL), showing a sigmoidal response. The curve was fitted using a logistic regression model to interpolate unknown sample concentrations. The standard curve demonstrated a strong correlation between fluorescence intensity and miR‐122 concentration. The mean of three independent measurements per sample was used for analysis. There was negligible cross‐reactivity between the miR‐122 beads for other analytes present in serum samples. Intra‐assay precision was 11.3%, and interassay precision was 10.3%. Accuracy was 109.3%. Sensitivity: lower limit of quantification (LLOQ) 20 pg/mL and lower limit of detection (LoD) 10 pg/mL.

In the patient group, the median miR‐122 concentration was significantly higher (233.4 pg/mL, IQR: 74.5–363.4) compared to the control group (15.2 pg/mL, IQR: 9.4–24). The range of miR‐122 concentration varied from 2.2 to 67.2 pg/mL in controls and from 17.5 to 1376.9 pg/mL in patients. Statistical analysis using the Mann–Whitney *U* test confirmed a significant difference between the two groups (*p* < 0.0001) (Figure 2B), supporting the potential role of miR‐122 as a biomarker for liver injury.

ROC analysis demonstrated that miR‐122 could distinguish DILI patients from healthy controls, with an AUC of 0.96 (95% CI: 0.91–1). At a cut‐off of 29.7 pg/mL, sensitivity was 92.6% (95% CI: 76.6% to 98.7%) and specificity was 89.4% (95% CI: 68.6% to 98.1%) (Figure 2C). Spearman correlation showed a moderate positive association between miR‐122 and ALT (*r* = 0.56, 95% CI: 0.056–0.84, *p* = 0.031) (Figure 2D).

## 4 DISCUSSION

This study presents data supporting the measurement of miR‐122 in serum using DCL which represents an alternative to quantitative PCR. In recent years, miR‐122 has emerged as a promising biomarker for liver injury, particularly in the context of paracetamol toxicity. Multiple studies have highlighted the diagnostic and monitoring potential of circulating miRNAs in liver damage.^17^, ^18^ In particular, miR‐122 correlates with the extent of liver damage, making it a potential diagnostic tool in clinical settings.^19^ Using a multiplex bead‐based system readily available in hospital laboratories, our results demonstrate the feasibility of quantifying miR‐122 in paracetamol overdose and highlight its potential for clinical use, aligning with previous studies that support the use of miRNAs as non‐invasive biomarkers to distinguish liver injury patients from healthy controls. Given the consistency of findings from previous studies, miR‐122 has been suggested as a clinically valuable biomarker for liver injury, with some reports indicating it may offer higher diagnostic accuracy than traditional liver enzyme markers.^6^ Therefore, miRNA‐based assays have promise as a tool for early detection and monitoring of liver injury, particularly in paracetamol overdose.

The direct quantification of miR‐122 by the novel bead‐based immunoassay that uses DCL technology offers several advantages over traditional PCR methods for measuring miR‐122, particularly in clinical settings. Unlike PCR, which requires extensive sample processing, RNA extraction and amplification steps, this assay enables direct and multiplexed quantification of miR‐122, thereby reducing analysis time, procedural complexity and overall costs. An important advantage is that multiplex technology is already available in many hospital laboratories, making it a feasible option for clinical translation without the need for additional infrastructure. Moreover, multiplex assays demonstrate a broader dynamic range and enhanced reproducibility compared to PCR‐based assays, making them well‐suited for large‐scale studies where consistency is critical.^20^, ^21^, ^22^ Since microsphere‐based multiplex assays do not rely on enzymatic amplification, they minimize amplification bias and variability associated with qPCR, potentially leading to more reliable and quantitative measurements. Given these advantages, the multiplex platform may provide a robust, high‐throughput and cost‐effective alternative for miR‐122 quantification, making it particularly useful for biomarker validation and clinical diagnostic applications. The cost of reagents per sample for the DCL assay is likely to be similar to that for RT‐qPCR, but the reduced number of steps and labour time required to run the assays is likely to make the DCL assay more cost‐effective.

This proof‐of‐concept study has several limitations. It involved a relatively small sample size and thus requires validation in a prospective multi‐centre study to confirm the findings. The time from sample application to result could be further reduced to enable more efficient clinical decision‐making in acute settings. The assay needs further optimisation to function with capillary blood which would allow point of care testing. The ability to diagnose liver injury on a finger prick of blood would allow testing immediately in hospital emergency departments and even prehospital (e.g., in an ambulance).

To enable a result to be available in a timeframe suitable for acute clinical decision making, rapid pre‐analytical steps will be needed to separate blood cells from serum.

In conclusion, the Luminex‐DCL miR‐122 assay represents a potential advance in the diagnosis and monitoring of liver injury as this assay can be performed using an established hospital laboratory platform and has, therefore, potential for widespread clinical adoption.

## AUTHOR CONTRIBUTIONS

**Samar Alzeer:** Writing—original draft; methodology; investigation; formal analysis; validation. **Antonio Marin‐Romero:** Methodology; investigation; formal analysis; validation. **Bárbara López‐Longarela:** Methodology; investigation; validation. **Juan J. Guardia‐Monteagudo:** Resources; investigation. **Francisco J. Lopez‐Delgado:** Resources; investigation. **Salvatore Pernagallo:** Conceptualization; methodology; investigation. **Juan J. Díaz‐Mochón:** Conceptualization; methodology; writing review and editing. **Mavys Tabraue‐Chavez:** Conceptualization; methodology; formal analysis; writing review and editing; supervision. **Kathleen Scullion:** Investigation. **James W. Dear:** Resources; conceptualization; methodology; formal analysis; writing review and editing; supervision.

## CONFLICT OF INTEREST STATEMENT

The authors declare the following financial interests which may be considered as potential competing interests: J.J.D.M. is a founder, shareholder and Director of DESTINA Genomics Ltd. S.P. is a shareholder of DESTINA Genomics Ltd. DESTINA Genomica. S.L. is a wholly owned subsidiary of DESTINA Genomics Ltd. DESTINA is interested in the exploitation of the technology developed here.

## Data Availability

Data are available on request from the authors.
